# The New Standardized Malaysian Healthy Eating Index

**DOI:** 10.3390/nu13103474

**Published:** 2021-09-30

**Authors:** Marhamah Jailani, Siti Masitah Elias, Roslee Rajikan

**Affiliations:** 1 Faculty of Science and Technology, Universiti Sains Islam Malaysia, Nilai 71800, Malaysia; marhamah.jailani@gmail.com; 2 Faculty of Health Science, Universiti Kebangsaan Malaysia, Kuala Lumpur 50300, Malaysia; roslee@ukm.edu.my

**Keywords:** healthy eating index, Malaysian population, least restrictive

## Abstract

Healthy Eating Index (HEI) is a diet quality measure that assesses the population’s compliance towards dietary guidelines. In Malaysia, diet quality measure, though existing, has some limitations in terms of application and relevance. This study aims to develop a new standardized Malaysian Healthy Eating Index (S-MHEI) that can measure the diet quality of all Malaysians regardless of their energy requirement level. The Malaysian Dietary Guidelines (MDG) 2010 and MDG for Children and Adolescents (MDGCA) 2013 were used as main references in developing the index components. In addition, the latest Malaysian Adults Nutrition Survey (MANS) and Adolescent Nutrition Survey (ANS) were also referred to ensure the relevance of the components selected. For adequacy components, the least restrictive method was used in setting the standard for the scoring system. Meanwhile, the scoring system for moderation components was built based on the Recommended Nutrient Intake (RNI) 2017. The new S-MHEI comprises of 11 components with a maximum total score of 100. The least restrictive method allowed the index to be used across energy requirement levels. However, the index will not be sensitive towards adhering to the specific recommended amount of intake—which in effect, made the index focus on measuring diet quality rather than diet quantity.

## 1. Introduction

The Healthy Eating Index (HEI) was first developed in 1995 to assess the overall diet quality of the American population by integrating nutrient needs and dietary guidelines in one single measure [1]. Since then, the index has been revised and improvised, and later was widely adapted by other countries to evaluate the population’s diet quality in terms of conformity towards the respective country’s dietary guidelines [2,3,4,5,6,7,8,9,10,11,12,13]. The validity and the reliability of the index has been proven by numerous studies [10,12,14,15,16,17,18,19,20,21,22]. In addition to the validity and reliability evaluation, researchers have also been expanding the use of the HEI to the studies in epidemiology, population and subpopulation monitoring and nutrition interventions [23,24,25,26,27,28,29,30,31,32,33,34,35,36,37]. Other possible uses of HEI include to scrutinize the connection between diet quality and diet cost [38], socio-demographic and economic indicator [39], and to evaluate diet quality in different levels of food environment (e.g., national level food supply, grocery stores and restaurant menus) [7,11,13]. 

In Malaysia, there were several attempts to develop a diet quality measure based on the Malaysian Dietary Guidelines (MDG) and Malaysian Food Pyramid (MFP), i.e., the Healthy Eating Index for Malaysian Adults (HEI-MA) [40], Malaysian Healthy Eating Index (MHEI) [41] and Malaysian Diet Quality Index (MDQI) [42]. The HEI-MA and MHEI were similar in terms of scoring system, where both adopted servings-based scoring criteria introduced by Kennedy et al., (1995) [1]. This method limited the use of the indices to only certain energy requirements included in the MDG 2010 (1500 to 2500 kcal). Furthermore, the criteria for minimum score for total fat and sodium (or natrium) in both indices were also derived based on the 85th percentile of the population intake as in the Malaysian Adults Nutrition Survey (MANS) 2003 which can be considered outdated and needed to be revised with the publication of the newer MANS 2014.

The only difference between these two indices was that MHEI included food variety as a component. Despite the importance of diet diversity in achieving a balanced diet, Waijers et al., had argued against the need of including diet variety as an index’s component as it may lead to a double-scoring problem [43]. In the case of MHEI, there were seven adequacy components of different food groups included, and the criteria of having the maximum score for the variety component was to have at least seven types of foods per day. Thus, obtaining a non-zero score for each adequacy component had actually implied diet diversity [11]. In contrast, a less diverse diet would be reflected through more zero scores which would accumulate into a lower total score. Therefore, even without the variety component, the combined score would eventually demonstrate diet variety in the consumption pattern.

On the other hands, MDQI was different from HEI in terms of component selection and scoring method. A food group will only be selected to be the index’s component if it passes the reliability and validity testing. In conducting the tests, MDQI used MANS 2003 Food Frequency Questionnaire (FFQ) data. This led to the same issue as HEI-MA and MHEI where the relevance of MDQI components need to be revised with the issuance of MANS 2014. Furthermore, MDQI used a bidirectional scoring system where the criteria for scoring consider both intake frequency and serving size. This method limits the DQI application in statistical analysis and further in diet–disease research [11].

Despite the limitations of the existing indices, numbers of studies were found using these indices as a measure of the population’s diet quality which reflects their importance [44,45,46,47,48]. This leads to the need for the development of one standard Malaysian HEI that can suit the needs of all energy requirement levels using the latest information available. Thus, this study aims to develop a new and more standardized MHEI that can be used to assess the population’s overall diet quality. The development of the index will be described thoroughly in this article.

The new standardized MHEI (S-MHEI) will be a very useful tool in assessing changes in Malaysians’ diet quality over time and the population adherence to the MDG provided by the Ministry of Health. This information will help the ministry in planning the intervention program, population monitoring and strategy in reducing the cases of malnutrition among Malaysians according to the food groups relevant. In addition, the index can be applied in a larger research area to have a deeper understanding about diet related diseases and demographic factors that influence poor dietary intake.

## 2. Materials and Methods

### 2.1. Method to Development the S-MHEI Components

To assess overall diet quality of Malaysian population, the components of the index were developed based on MDG 2010 [49] and Malaysian Dietary Guidelines for Children and Adolescent (MDGCA) 2013 [50]–the latest version of the MDG and MDGCA available. These guidelines compiled the science-based nutrition and physical activity recommendation with the aim to provide culturally sensitive dietary advice suited to the dietary needs of the various communities in Malaysia apart from physical activity recommendation. Diet-related recommendations were shortlisted and analyzed before being translated into the index components and divided into either adequacy or moderation type. The adequacy component refers to food group that was recommended to be consumed sufficiently while moderation component denotes food group that was advised to be taken within reasonable limits.

Since the MDG and the MDGCA are not being revised very regularly, the relevance of the components was further analyzed by looking at the population’s intake in Malaysian Adult Nutrition Survey (MANS) 2014 [51] and Adolescent Nutrition Survey (ANS) 2017 [52]. In the case where the survey result proves that the intake of the short-listed component has been satisfied by the population, that particular component will be taken out from the list so that the end result is very specific and focuses only on the dietary quality that needs to be improved. This additional analysis is important in reassessing the relevance of component type. For instance, if the intake level of an adequacy component is too high, the component type should be changed from adequacy to moderation to ensure the consumption of the component is within the standard of diet quality.

### 2.2. Method to Develop the S-MHEI Scoring Standard

This study adopted the least restrictive standard introduced by Guenther et al. (2008) [2] in setting the criteria for the scoring of population dietary intake. In this standard, the easiest-to-achieve recommendation among appropriate energy requirement level was chosen as the cut-off point for maximum score. As for the threshold value for minimum score, all adequacy components will take the value 0 which indicates no consumption while the moderation and optimal components will refer to the recommendations in the Recommended Nutrient Intake (RNI) 2017 [53] and the latest WHO guidelines (if existing) which were built based on clear evidence that specifies how high an intake must be to be considered high enough that it deserves the score 0.

Using this standard, the scores are expected to turn out higher than when the standard is not applied as it focuses more on identifying intakes that do not meet the recommendations [2] rather than being sensitive in terms of fulfilling specific amount of food for each energy requirement. In effect, the final score will give information on the level of diet quality achieved by the population instead of explaining how many people do not meet the precise amount of food that they are supposed to consume. 

In setting the standard, energy density approach was used where the recommended servings for each food component were converted into amounts per 1000 kcal for every energy requirement level. To obtain the serving size of the food components for every energy requirement level, computer simulation method was used and 10,000 data sets were generated based on the amount of carbohydrate, protein and fat in each food component as outlined in MDG2010. Any data set that is outside the recommended percentage of macronutrient contribution towards Total Energy Intake (TEI) as described in RNI 2017 (carbohydrates 50–65% TEI, fat 25–30% TEI and protein 10–20% TEI) [53] were eliminated. In the case that a specific micronutrient or macronutrient was chosen as one of the index’s components, the recommendations were expressed in either milligrams (mg) or percentage of daily TEI. The elimination of simulated dietary intake that falls outside the acceptable percentage will mimic a more plausible and healthy diet intake for each calorie level.

## 3. Results and Discussion

### 3.1. Components of the S-MHEI

The MDG 2010 and MDGCA 2013 consists of key messages covering the recommendations on food variety, body weight, physical activity, food and beverages consumption, flavors, water consumption, breastfeeding practice, food safety and food labels. On the first stage, key messages that are not relevant were excluded (i.e., body weight, physical activity, breastfeeding practice, food safety and food labels). Food variety recommendation was also removed from being part of the index component due to the reasons presented in the previous subsection.

Findings of MANS 2014 showed that Malaysian adults have met the recommended intake of plain water in terms of its amount and frequency [51]. Apart from that, plain water is also calorie-free which made it irrelevant to be included in the index. Thus, water was also eliminated from being part of the index component. It is also important to note that both MANS 2014 and ANS 2017 highlighted the concern on the increasing intake of sugar among Malaysian adults and adolescents. Therefore, added sugar was included as one of the index components even though there are no specific key messages on the recommended amount of intake in both the MDG 2010 and MDGCA 2013.

The remaining key messages were then scrutinized and developed into 11 components that reflect both the MDG 2010 and MDGCA 2013 recommendations on healthy food consumption. The 11 components were further grouped into three categories according to their recommendation type, i.e., adequacy, optimal or moderation. The components, their type and the recommendations from the MDG 2010 and MDGCA 2013 from which the components were derived are presented in the following Table 1.

### 3.2. S-MHEI’s Score and Interpretation

For most of the index’s components, a maximum of 10 points are assigned when the specific criteria are fulfilled. However, for ‘total grains’ (which include rice, other cereal products and tubers; and ‘whole grain’, the maximum score assigned for both components is five to avoid overlapped scoring since both components are from the same food group. In effect, the whole index has a total score of 100. A dietary intake score of more than 80% shows good diet quality while a score between 51 and 80% indicates the diet quality needs an improvement, while a total score below 51% is considered poor diet quality [54]. 

### 3.3. Standard for Scoring Adequacy Component

For adequacy components, the minimum score of zero is assigned when the component is not consumed at all, and the maximum score will be given when the component intake is equal or higher than the cut-off value. The score of intakes between zero and the cut-off value are prorated linearly and calculated as below:

(1) $$Score=\frac{The\;reported\;intake}{The\;cut\;off\;value}\times The\;maximum\;score$$

**Figure 1:**
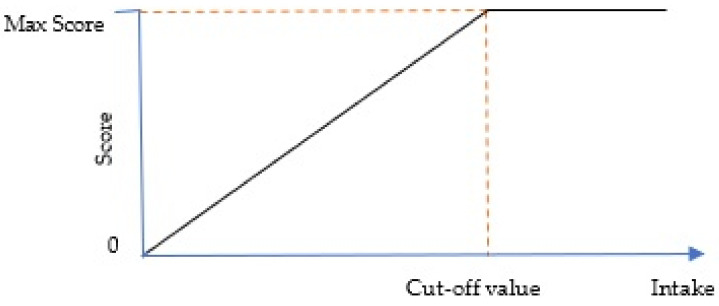
Relationship between score and adequacy component intake.

The following Figure 1 illustrates the relationship between the score and the intake of adequacy component.

The cut-off value for adequacy components is the lowest recommended amount ‘per 1000 kcal’ for daily energy intake between 1200 to 3000 kcal. When an energy density approach was applied, the amount appeared to be quite similar across energy levels (Table 2) and not increasing linearly as when they were not expressed in ‘per 1000 kcal’ unit. Thus, by applying the least restrictive method in determining the cut-off value, the value chosen still meets the needs of people who have higher energy requirements since the higher calorie patterns had the same nutrient goals [2]. For example, in Table 2, the lowest recommended amount of total grains for daily energy intake between 1200 to 3000 kcal grains is 1.4 servings per 1000 kcal. This means that for someone who needs 2500 kcal, he must consume at least (2500 kcal × 1.4 servings/1000 kcal) 3.5 servings daily to get the maximum score while someone who needs 3000 kcal must consume at least (3000 kcal × 1.4 servings/1000 kcal) 4.2 servings daily to achieve the maximum score. 

Energy requirements lower than 1200 kcal are not considered since they usually aimed only at children between 2 to 3 years old whose recommended nutrient intake is too low compared to the rest of the population, even when expressed on a density basis. 

### 3.4. Standard for Scoring Moderation Components

For the moderation component, zero will be assigned if the component consumption is above the threshold value and the maximum points will be given if the intake is equal to or lower than the cut-off value. The score between the cut-off value and the threshold value will be awarded proportionately as the calculation below:

(2) $$Score=\frac{\left(The\;threshold\;value-The\;reported\;intake\right)}{\left(The\;threshold\;value-The\;cutoff\;value\right)}\times The\;maximum\;score$$

**Figure 2:**
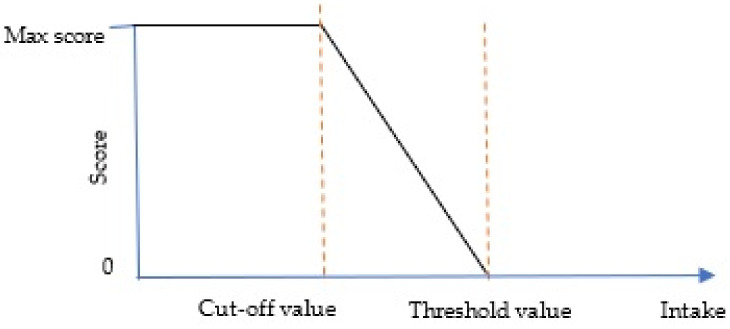
Standard for scoring moderation components.

Determining the threshold value for the moderation component is quite tricky since there is no clear scientific evidence that specifies how high an intake must be to be considered high enough that it deserves the score 0 [2]. In the case where a standard is set to be high enough that a large proportion of the population get the minimum score of zero, overtime changes and difference among individuals and groups will be hard to detect at the low end of the scoring range [2]. Figure 2 illustrates the relationship between the score and the intake of moderation components.

#### 3.4.1. Sodium

For sodium, WHO recommends less than 2000 mg sodium per day for adults and the amount shall be adjusted downward based on the energy requirements of children relative to those of adults [55]. Meanwhile, the RNI 2017 recommendation on sodium intake for children aged 4 years old to adults is between 1200 to 1500 mg per day; and 2300 mg was set as the highest upper limit among all ages since it has the lowest observed adverse effect level (LOAEL). Findings from ANS 2017 found that, 75.1% of adolescents exceeded the NPANM III target, 19.2% achieved them and 5.7% took less than 75% of the recommended intake of sodium. The median sodium intake as a percentage of the recommended nutrient intake among adolescents (1500 mg sodium was recommended for adolescent of age 13–17 years old) was 179.65%, which is equivalent to 2694.73 mg. On the other hand, MANS 2014 showed that the median sodium intake among Malaysian adults was about 1935 mg/day.

The concern with high salt intake as raised in MDG 2010 was based on the evidence of an association between dietary salt intake and blood pressure which has been regarded by WHO as high-quality evidence [55]. The recent National Health and Morbidity Survey (NHMS) 2019 also reported the high prevalence of hypertension among Malaysian adults was still high and only slight reduction was recorded from the year 2015 (30.3%) to the year 2019 (30.0%). This progress is quite insignificant given that Malaysia, as a WHO member state, has agreed to bring down the global population salt consumption by 30% by year 2025 [56]. 

Therefore, 2300 mg will be set as the threshold value for the minimum score, zero, due to the concern on the LOAEL. Since 80% marks a good diet quality, 2000 mg of sodium intake is set to get the score of eight and as a result, 1925 mg of sodium intake will mark the cut-off value for maximum point of 10, which satisfies the recommendations of WHO. Even though the cut-off value exceeds RNI 2017 recommendation, it is still acceptable since the main concern in measuring diet quality is more to observe the population who do not adhere to dietary recommendations rather than strictly abiding by the quantity suggested. In other words, we are more interested in the number of populations who get the minimum score compared to number of populations who do not reach the maximum score. The following Table 3 summarizes the score and level of sodium intake.

#### 3.4.2. Added Sugar

In the case of added sugar, MDG 2010 quoted the Dietary Reference Intake (DRI) committee of the Institute of Medicine (IOM) (2002) in setting the upper limit of sugar intake which is 25% of TEI [49,57]. On the other hand, WHO recommends an intake of free sugar for both adults and children to be less than 10% of TEI, and further reduction to below 5% of TEI is suggested [58]. 

The recommendation on the limitation of added sugar consumption is based on the suggestion of its association with obesity and dental caries [49,58]. Besides that, apart from calories, added sugar contains no vitamins and minerals [49]. Thus, over consumption of sugar could replace micronutrient-dense food from diet and lead to greater risk of vitamin and mineral deficiency [49]. In terms of population intake, ANS 2017 showed a tremendous increase in the median sugar intake among adolescents, i.e., from 29.53 g in 2012 to 40.71 g in 2017. However, 57.2% of the adolescents were found to satisfy the recommended consumption of free sugar to be less than 10% of TEI. On the other hand, MANS 2014 recorded the mean intake of sugar of 25.52 g/day for Malaysian adults with a prevalence of 55.9%. Based on NHMS 2019, 30.4% of adults in Malaysia were found to be overweight while 19.7% were found to be obese, which increased from the survey conducted in 2015 (30% overweight and 17.7% obese). The same case goes for the children aged 5–17 years old, where 15% of them were found overweight while 14.8% were obese, an increase of 2.9% compared to findings from NHMS 2015 (11.9% prevalence of obesity among children).

Based on the recommendations, the survey reports on sugar intake among the population and the prevalence of obesity among Malaysians, 25% of TEI is set as the threshold for added sugar while the cut of value for the maximum score is set to be less than 5% of TEI. Table 4 summarizes the added sugar intake for all scores point from 0 to 10.

### 3.5. Standard for Scoring Optimal Components

**Figure 3:**
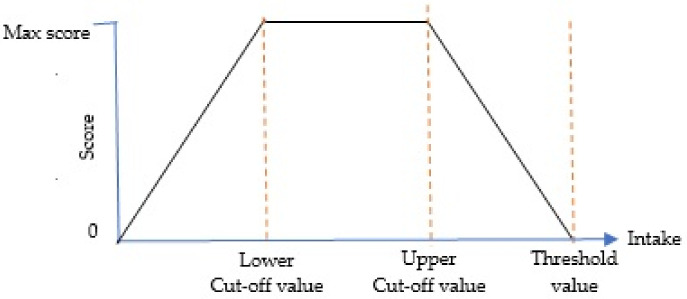
Standard for scoring optimal components.

For optimal component, zero will be assigned in two conditions. An individual will get a score of zero if there is no intake of the component at all, or, if the intake is higher than the threshold value. On the other hand, the maximum points will be given if the intake is between the recommended intake range which is marked by lower and upper cut-off values. The score between zero intake and the lower cut-off value is calculated using Formula (1) while the score for intake between the upper cut-off value and the threshold value is awarded proportionately as the calculation in Formula (2). Figure 3 illustrates the relationship between the intake of optimal component and the score for better comprehension. 

#### Total Fat (TF)

Total fat is put under optimal component since it is recommended to be consumed within a specified range. Fat is important in the human diet as it helps in physiological and structural function, growth and development (especially during infancy), the digestion process and survival during limited food availability [49,50,53]. However, excess intake of high fat diet coupled with low energy expenditure may lead to obesity [49]. In addition, a rise in plasma triglyceride—an independent risk factor of atherogenesis—was also found to be contributed by high fat loaded diet [59].

For total fat (TF), both the MDG 2010 and MDGCA 2013 recommended the limit on daily intake to be less than 30% of TEI—which is in line with the recommendation of WHO in avoiding unhealthy weight gain [49,50,60]. However, the lower limit for adults was set at 20% TEI while the lower limit for children is 25% TEI. Revision on the limit by RNI 2017 raised the minimum recommended intake for adults from 20 to 25% TEI and introduced 35% TEI as an upper safe limit for an active adult [53]. In addition, RNI 2017 also proposed 25–35% TEI as the new range of children and teenagers’ total daily fat intake [53]. 

MANS 2014 reported the median total fat intake among Malaysian adults as 29% TEI, which is aligned with the recommendations discussed previously [51]. However, NHMS 2015 recorded that 17.7% of the Malaysian population were obese and 30% were overweight which in total make almost half of the population [61]. Despite the unhealthy trend in the population’s weight, the findings of NHMS 2015 do not support the conclusive association between obesity and fat intake [53]. However, according to ANS 2017, the prevalence of inadequate TF intake among adolescents sums up to 53.1% where 42.2% of them were reported to have more than 35% TEI contributed by fat while the other 10.9% had less than 25% TEI contributed by fat. The excess and deficiency in daily TF intake among adolescents are something worrisome since these unhealthy habits might develop into risk in the future—thus explaining the significance of the existence of TF as one of the S-MHEI components. 

The method of even distribution between the interval of crucial score (0, 5, 8 and 10) will be used here. Considering the recommendations by MDG 2010, MDGCA 2013 and RNI 2017, 20 and 35% TEI will both correspond to score of eight, which is the minimum mark to show good TF intake, while 25 and 30% TEI will mark the lower and upper cut-off values for maximum score of 10. Consequently, the threshold value for minimum score will be set at 0 and 55% TEI. Even though it is nearly impossible to record a daily TF intake of 0% TEI, it will be maintained in the scoring standard to emphasize the importance of having optimal TF intake and at the same time capture any changes in TF deficiency. This will allow the index to give a low score whenever the intake of TF is below the lower cut-off value. The following Table 5 summarizes the TF intake for all scores point from 0 to 10.

### 3.6. The New Standardized Malaysian Healthy Eating Index (S-MHEI)

The summary of the new standardized Malaysian Healthy Eating Index as a whole is described in Table 6 as follows.

### 3.7. Strengths and Limitations of S-MHEI

In general, HEI is a simple diet quality measuring tool which is easy to apply and has a straightforward explanation compared to other similar tools. In terms of this new standardized MHEI, almost all components of the existing diet quality indices were retained. In addition to that, whole grain and added sugar were introduced to address the suboptimal intake within the population and the current necessities in terms of the prevalence of diet-related diseases, apart from highlighting the recommendations included in the MDG 2010 which were not brought forward before. 

Furthermore, in setting the scoring criteria, the density standard was used instead of the serving-base standard in order to widen the use of the index to all levels of energy requirements rather than just for adults. Besides that, the least restrictive (easiest to achieve) of the recommended amounts of food to consume was chosen in determining the threshold and cut-off value for minimum and maximum score so that this new index can be more specific in identifying intakes that do not meet the recommendation rather than being sensitive towards adhering to the recommended intake. As a result, this index will identify some but not all intakes that do not meet individual recommendations. However, all the errors will be in the same direction and the index will separate between diet quality from diet quantity. 

Despite the concern on the excess intake of saturated fat (SF) on health, TF was chosen over SF to be one of the components due to limitations in terms of the SF database and assessment method. However, whenever the complete database is available and the assessment method is well developed, this index shall be revised and SF is highly recommended to be included as one of moderation components. 

It is also important to note that this index is not suitable for children younger than age two, pregnant women and diseased individuals due to their special dietary requirements such as breast milk, additional supplements, etc. Thus, for these groups, another specific HEI can be developed.

## 4. Conclusions

The new developed S-MHEI assess the diet quality in terms of conformity to the dietary guidelines specified for the Malaysian children, adolescents and adults’ needs. The assessment is focused on the food and beverages consumption and nutrients gained from them as it emphasizes balance among food groups rather than the quantity consumed. This can be seen from the same maximum score assigned for all food groups. In effect, the index highlighted the food group to encourage and to reduce. This feature is also important as it accommodates the variety of eating patterns in Malaysian multiracial culture. The least restrictive approach employed by this index ensures that the criteria for maximum score are the easiest to achieve despite the difference in age and sex which is the main difference of the index with the existing ones.

## Figures and Tables

**Table 1:** S-MHEI’s components and related MDG2010 and MDGCA2013 recommendations.

No	Component	Type ^a^	Recommendation from MDG2010	Recommendation from MDGCA2013
1	Total grains	A	Consume at least four servings of cereal foods daily	Ensure an adequate intake of cereal and cereal based foods according to age
2	Whole grains	A	Choose at least half of your grain products from whole grains	Ensure that at least half of daily cereal intake includes whole grain
3	Fruits	A	Eat at least 2 servings of fruits a day	Eat variety of fruit and vegetables every day. Eat adequate amount of fruits and vegetables every day
4	Vegetables	A	Eat at least 3 servings of vegetables a day	
5	Fish	A	Choose fish more frequently; if possible, daily	Eat fish daily
6	Meat, poultry and eggs	A	Consume meat, poultry and egg moderately	Consume meat, poultry and egg moderately
7	Legumes and nuts	A	Consume legumes daily. Include nuts and seeds in weekly diet	Consume legumes daily Include nuts and seeds in weekly diet
8	Milk and milk products	A	Consume milk and milk product everyday	Consume 2 to 3 servings of milk and milk products everyday
9	Total Fat	O	Minimize the use of fat in food preparation in order to keep total daily fat intake between 20 to 30% energy	Limit total daily fat intake to 25 and 30% of energy
10	Sodium	M	Limit salt intake to 1 teaspoon a day Reduce consumption of highly salted foods and condiments	Limit intake of salt and sauce
11	Sugar	M	Consume food and beverages low in sugar.	Consume food and beverages low in sugar.

^a^ A refers to adequacy component; O refers to optimal components; M refers to moderation component.

**Table 2:** Recommended servings according to food group (adequacy component), expressed per 1000 kcal.

Food Group	Calorie Level (kcal)																				
	1000	1100	1200	1300	1400	1500	1600	1700	1800	1900	2000	2100	2200	2300	2400	2500	2600	2700	2800	2900	3000
Total grains ^1^	1.0	1.5	1.4	1.4	1.6	1.7	1.9	2.1	2.3	2.4	2.6	2.7	2.9	3.0	3.0	3.0	3.0	3.0	3.0	2.9	2.9
Whole grain ^1^	0.5	0.8	0.7	0.7	0.8	0.9	1.0	1.1	1.2	1.2	1.3	1.4	1.5	1.5	1.5	1.5	1.5	1.5	1.5	1.5	1.5
Fruits ^2^	1.5	1.4	1.5	1.5	1.5	1.5	1.3	1.2	1.2	1.1	1.1	1.0	0.9	0.9	0.9	0.9	1.0	1.1	1.1	1.1	1.2
Vegetables	1.5	1.4	1.3	1.3	1.3	1.2	1.3	1.3	1.4	1.4	1.5	1.5	1.6	1.8	1.7	1.6	1.6	1.4	1.4	1.2	1.2
Fish ^3^	0.6	0.6	0.6	0.6	0.7	0.6	0.6	0.6	0.6	0.5	0.5	0.5	0.5	0.5	0.5	0.5	0.5	0.5	0.5	0.4	0.4
Meat, poultry and eggs ^4^	0.3	0.4	0.4	0.5	0.5	0.6	0.6	0.7	0.7	0.7	0.7	0.7	0.8	0.8	0.8	0.8	0.8	0.8	0.8	0.8	0.8
Legumes and Nuts ^5^	0.9	0.9	0.7	0.8	0.7	0.7	0.6	0.6	0.6	0.5	0.5	0.5	0.4	0.4	0.4	0.4	0.4	0.4	0.4	0.4	0.4
Milk and milk products ^6^	2.3	1.8	1.7	1.6	1.5	1.4	1.4	1.3	1.2	1.2	1.1	1.1	1.0	1.0	1.0	1.0	0.9	0.9	0.9	0.9	0.9

^1^ based on 30 g carbohydrate per serving; ^2^ based on 15 g carbohydrate per serving; ^3^ based on 14 g protein per serving; ^4^ based on 14 g protein per serving; ^5^ based on 7 g protein per serving; ^6^ based on 7 g protein per serving.

**Table 3:** Summary of score correspond to Sodium intake (mg).

Score	0	1	2	3	4	5	6	7	8	9	10
Intake (mg)	≥2300.0	2265.5	2225.0	2187.5	2150.0	2112.5	2075.0	2037.5	2000.0	1962.5	≤1925.0

**Table 4:** Summary of score correspond to Added Sugar intake (% of TEI).

Score	0	1	2	3	4	5	6	7	8	9	10
Intake (% of TEI)	≥25	23	21	19	17	15	13	11	9	7	≤5

**Table 5:** Summary of score corresponding to total fat intake (% of TEI).

Score	0	1	2	3	4	5	6	7	8	9	10
Intake (% of TEI)	≥55.0	52.5	50.0	47.5	45.0	42.5	40.0	37.5	35.0	32.5	25.0–30.0
	0.0	2.5	5.0	7.5	10.0	12.5	15.0	17.5	20.0	22.5	

**Table 6:** The new standardized Malaysian Healthy Eating Index (S-MHEI).

No	Component	Type	Max Score	Criteria for Min Score (0)	Criteria for Max Score
1	Total grains	A	5	0 servings/1000 kcal	1.4 servings/1000 kcal
2	Whole grains	A	5	0 servings/1000 kcal	0.7 servings/1000 kcal
3	Fruits	A	10	0 servings/1000 kcal	0.9 servings/1000 kcal
4	Vegetables	A	10	0 servings/1000 kcal	1.2 servings/1000 kcal
5	Fish	A	10	0 servings/1000 kcal	0.4 servings/1000 kcal
6	Meat, poultry and eggs	A	10	0 servings/1000 kcal	0.4 servings/1000 kcal
7	Legumes and nuts	A	10	0 servings/1000 kcal	0.4 servings/1000 kcal
8	Milk and milk products	A	10	0 servings/1000 kcal	0.9 servings/1000 kcal
9	Total Fat	O	10	0 or ≥55% of TEI	25–30% of TEI
10	Added Sugar	M	10	≥25% of TEI	≤5% of TEI
11	Sodium	M	10	≥2300 mg	≤1925.0 mg

## Data Availability

Data available on request. The data presented in this study are available on request from the corresponding author.
